# Inflammation reduces mechanical thresholds in a population of transient receptor potential channel A1-expressing nociceptors in the rat

**DOI:** 10.1111/j.1460-9568.2008.06256.x

**Published:** 2008-06

**Authors:** James P Dunham, Sara Kelly, Lucy F Donaldson

**Affiliations:** Department of Physiology and Pharmacology, School of Medical Sciences, University Walk, University of BristolBristol BS8 1TD, UK

**Keywords:** complete Freund's adjuvant, inflammation, mechanonociception, pain, primary afferent, transient receptor potential channel A1

## Abstract

Inflammatory hypersensitivity is characterized by behavioural reductions in withdrawal thresholds to noxious stimuli. Although cutaneous primary afferent neurones are known to have lowered thermal thresholds in inflammation, whether their mechanical thresholds are altered remains controversial. The transient receptor potential channel A1 (TRPA1) is a receptor localized to putative nociceptive neurones and is implicated in mechanical and thermal nociception. Herein, we examined changes in the properties of single primary afferents in normal and acutely inflamed rats and determined whether specific nociceptive properties, particularly mechanical thresholds, are altered in the subpopulation of afferents that responded to the TRPA1 agonist cinnamaldehyde (TRPA1-positive afferents). TRPA1-positive afferents in normal animals belonged to the mechanonociceptive populations, many of which also responded to heat or capsaicin but only a few of which responded to cold. In acute inflammation, a greater proportion of afferents responded to cinnamaldehyde and an increased proportion of dorsal root ganglion neurones expressed TRPA1 protein. Functionally, in inflammation, TRPA1-positive afferents showed significantly reduced mechanical thresholds and enhanced activity to agonist stimulation. Inflammation altered thermal thresholds in both TRPA1-positive and TRPA1-negative afferents. Our data show that a subset of afferents is sensitized to mechanical stimulation by inflammation and that these afferents are defined by expression of TRPA1.

## Introduction

Inflammation is associated with altered pain perception, such as pain on innocuous stimulation (allodynia) and enhanced pain to noxious stimulation (hyperalgesia). The transient receptor potential channel A1 (TRPA1) is expressed in a population of primary sensory neurones and is co-expressed with putative markers of nociceptive afferents, such as transient receptor potential channel V1 (TRPV1), substance P and calcitonin gene related peptide (Story *et al.*, 2003; Kobayashi *et al.*, 2005). The functional properties of the population of TRPA1-expressing afferents have yet to be defined.

It has been hypothesized that TRPA1 could be a mechanically (Corey *et al.*, 2004; Nagata *et al.*, 2005; Kwan *et al.*, 2006; Hill & Schaefer, 2007; but see also Bautista *et al.*, 2006), cold (Story *et al.*, 2003; Sawada *et al.*, 2007; but see also Jordt *et al.*, 2004; Nagata *et al.*, 2005; Munns *et al.*, 2006) and/or chemically (Bandell *et al.*, 2004; Jordt *et al.*, 2004; Bautista *et al.*, 2006; McNamara *et al.*, 2007) activated channel. TRPA1 is activated by a wide range of natural and synthetic compounds, including many of the pungent compounds in spices, e.g. allyl isothiocyanate (mustard oil) and cinnamaldehyde (cinnamon oil), and environmental irritants and other exogenous and endogenous aldehyde-containing compounds (Bandell *et al.*, 2004; Jordt *et al.*, 2004; McNamara *et al.*, 2007; Trevisani *et al.*, 2007). TRPA1 agonists applied to the skin in humans cause spontaneous pain and heat and mechanical hyperalgesia (Namer *et al.*, 2005), whereas in rodents, intraplantar or topical cinnamaldehyde causes acute nocifensive behaviour, mechanical allodynia and heat hyperalgesia (Bandell *et al.*, 2004; Proudfoot *et al.*, 2006).

Altered nociception in the acute inflammation caused by formalin has recently been reported to be mediated through TRPA1-expressing afferents (McNamara *et al.*, 2007). In inflammation, TRPA1 is also activated or sensitized by stimulation of endogenous pro-inflammatory receptors, such as the bradykinin B2 receptor or nerve growth factor receptor, potentially contributing towards altered nociception in acute inflammation (Bandell *et al.*, 2004; Jordt *et al.*, 2004; Diogenes *et al.*, 2007). Intrathecal antisense TRPA1 reduces inflammation-associated cold hyperalgesia but not mechanical allodynia (Obata *et al.*, 2005). In contrast, pharmacological inhibition of TRPA1 affects both cold and mechanical behavioural changes in more acute (hours) inflammation (Petrus *et al.*, 2007). Furthermore, inflammation increases the numbers of neurones expressing TRPA1 mRNA (Obata *et al.*, 2005).

These data suggest that TRPA1 plays a role in the functional changes seen in primary afferent neurones in inflammation. The multiple sensory changes evoked by TRPA1 agonists have led to the proposal that TRPA1 could be a polymodal nociceptive transducer (Kwan *et al.*, 2006). The functional properties of TRPA1-expressing afferents have yet to be reported in either normal animals or in inflammation. Nociceptors are known to alter their functional properties in inflammation, with altered thresholds and the development of ongoing spontaneous activity (Guilbaud *et al.*, 1985; Schaible & Schmidt, 1985; Grigg *et al.*, 1986; Djouhri *et al.*, 2006). We therefore hypothesized that, as a putative polymodal transducer, TRPA1 would be principally found in polymodal nociceptors and that the properties of TRPA1-expressing afferents would be altered in acute inflammation.

## Materials and methods

### Animals

All experiments were carried out in accordance with the UK Animals (Scientific Procedures) Act, 1986 and associated guidelines. Male Wistar rats (250–350 g) were used in all experiments. Animals were given access to food and water *ad libitum* and housed in accordance with UK Home Office regulations. A total of 84 animals were used in these studies.

### CFA-induced inflammation

Animals were briefly anaesthetized using 4% halothane in O_2_ (2 L/min) and maintained areflexive on 2% halothane in O_2_. Once areflexia was achieved, 50 μL of complete Freund's adjuvant (1 mg.mL^-1^) was injected into each of two sites at either side of the tibiotarsal joint (total injected volume 100 μL) for subsequent neurophysiological recording of cutaneous afferents (*n*= 8 rats). Local injections around the tibiotarsal joint cause both a pronounced arthritis and extensive cutaneous inflammation encompassing the majority of the foot (Donaldson *et al.*, 1993). Furthermore, animals exhibit both mechanical allodynia and thermal (heat) hyperalgesia (Donaldson & Chillingworth, 2007). After complete Freund's adjuvant (CFA) injection, rats were returned to the home cage, allowed to recover and monitored daily for joint swelling.

### Neurophysiological and pharmacological characterization of primary afferents

Electrophysiology was performed on two groups of animals, those that had received an injection of CFA injection around the ankle joint 3 days previously (*n*= 8) and uninjected naive animals (*n*= 64).

Animals were anaesthetized (sodium pentobarbital, 60 mg/kg i.p.) and maintained deeply anaesthetized and areflexive (sodium pentobarbital, 20 mg/kg/h i.v.). The trachea was cannulated to maintain the airway and the external jugular vein and an artery (external carotid and/or femoral) were cannulated for anaesthetic administration, blood pressure monitoring and/or drug delivery. The body temperature was maintained within physiological limits by means of a feedback-controlled heater and rectal thermister. At the end of all experiments, rats were killed by an overdose of sodium pentobarbital (60mg).

The right saphenous nerve was exposed mid-thigh via an incision from the inguinal fossa to a point just distal to the knee joint and was isolated from the surrounding tissue. A pool of warmed paraffin oil was made of the surrounding skin to prevent dehydration and, following removal of the epineurium, fine filaments of the saphenous nerve were teased to enable differential recording of neuronal activity via bipolar platinum wire electrodes. Filaments were teased until they contained a small number (often ≤ 2 but occasionally 3) of identifiable afferents. Multiple filaments were studied in each animal in order to reduce the number of animals used. When collecting data from several filaments in the same animal, only units with non-overlapping receptive fields were characterized, so as to avoid sensitization by previously applied stimuli. Action potentials were amplified and passed through an analogue-to-digital converter (1401, Cambridge Electronic Design, Cambridge, UK). Spikes were recorded and analysed using spike 2 v5 software (Cambridge Electronic Design).

The receptive fields of primary afferent fibres were initially identified by monopolar electrical stimulation of discrete (1 mm diameter) areas of the hindpaw (up to 100 V, 0.5 ms duration) (Kress *et al.*, 1992) and conduction velocities were determined. C- and A-fibre conduction velocity boundaries were determined from saphenous nerve compound action potentials recorded in the same region of the saphenous nerve, performed in animals of the same sex and weight as those used for subsequent experiments. Using compound action potentials we determined that, in our preparation, the conduction velocities of C-fibres were always < 1 m/s and those of A-δ-fibres were between 5 and 15 m/s. All of the more rapidly conducting cutaneous afferents were classed as Aβ-fibres but, as histological studies suggest that TRPA1 is found in the small, more slowly conducting afferents (Jordt *et al.*, 2004; Kobayashi *et al.*, 2005; Obata *et al.*, 2005), Aβ units were not studied further.

Following the identification of the primary afferent fibre receptive field and conduction velocity measurements, the mechanical and thermal thresholds of the afferent neurones (units) were determined. The mechanical thresholds were determined using calibrated von Frey hairs (Linton Instruments, Norfolk, UK) as has been reported in many previous studies in both *in vivo* and *in vitro* preparations (Kumazawa & Perl, 1977; Lynn & Carpenter, 1982; Leem *et al.*, 1993; Koltzenburg *et al.*, 1999). Filaments were applied to the most sensitive region of the receptive field for approximately 3 s. The weight of the lowest force filament that reproducibly evoked activity was defined as the threshold of the unit, as previously described using a similar method (Dina *et al.*, 2004). It should be noted that hand-held von Frey hairs give an approximation of the mechanical thresholds of primary afferent units as the application of a range of hairs exerts incremental, discrete forces rather than a continuous force on the receptive field. Units that did not respond to stimuli > 180 g were not included in the analysis as it could not be determined whether these units were ‘silent’ nociceptors (Michaelis *et al.*, 1996) or whether the receptive field could not be located. In some cases units were also tested with gentle moving mechanical stimulation (brushing) to define unit type.

Thermal thresholds were determined using a Peltier device (built in-house, surface area ca. 8 mm^2^) to heat or cool the cutaneous receptive field of the unit under study. The Peltier was placed on the receptive field in contact with the skin. In the event of this evoking activity in any low-threshold afferents in the filament under study, the Peltier was repositioned to eliminate this before thermal stimulation began, to ensure that thermal responses were not contaminated with low-threshold afferent activity. The Peltier temperature was ramped from a holding temperature of 30–5°C (∼2°C/s) and then, after an interstimulus interval of no less than 2 min, from 30°C to 50°C (∼5°C/s). Occasionally the cold stimulus was reduced to 0°C if a clear cold response was not evident at 5°C. C-cold units were initially identified by their characteristic bursting discharge at room temperature (20-25°C) (Hensel, 1981). Using the same thermal stimuli, C-cold units responded vigorously to cold stimulation and were inhibited by heat stimulation (Hensel, 1981). The Peltier temperature at which afferent firing began was taken as the heat or cold threshold for each unit.

‘Classical’ cold-responsive fibres (Hensel & Zotterman, 1951) were also identified in these experiments. The temperature at which ongoing activity in these fibres begins is above that of the normal cutaneous temperature (28–30°C) and so these afferents were easily identified by their ongoing activity. This stimulus/response pattern meant that the cold activation threshold (i.e. the temperature at which firing began in response to cooling) for these afferents could not be easily determined. In addition, none of these units were TRPA1-positive and therefore these units were also excluded from further threshold analysis.

If a unit responded to at least two out of noxious mechanical, chemical, heat and cold stimulation it was classified as a polymodal nociceptor. Units that only responded to mechanical stimuli were classified as nociceptive if the threshold was greater than 1 g (Lynn & Carpenter, 1982).

### Close intra-arterial drug administration

For close intra-arterial drug administration, the left femoral artery was cannulated in the groin and the cannula advanced ca. 25 mm to the bifurcation of the descending aorta. In experiments in which units were not seen to respond to intra-arterial drug administration, the position of the cannula at the aortic bifurcation was confirmed visually at the end of the experiment. Agonists were administered in a 100 μL bolus (10% ethanol, 10% Tween 80, 80% saline) washed into the hindpaw with 400 μL of heparinized saline (50 U/mL). Antagonists were delivered in the same manner 60 s prior to agonist delivery and a minimum of 5 min was left between agonist/antagonist doses. In experiments in which afferent responses to capsaicin (10 μm) and/or cinnamaldehyde (80 mm) were determined, agonists were injected as described with a minimum of 5 min between injections. The elicited activity was then quantified as detailed in the Data and statistical analysis section below. Units that responded to both, or either, agonists were counted and the overlap in responsive populations calculated. Preliminary studies showed that the cinnamaldehyde-evoked response did not desensitize after three consecutive injections. No more than three injections were made per animal.

### TRPA1 expression in DRG in acute inflammation

Animals were briefly anaesthetized using 4% halothane in O_2_ (2 L/min) and maintained areflexive on 2% halothane in O_2_. Once areflexia was achieved, 100 μL of CFA (1 mg/mL, Sigma-Aldrich, Dorset, UK) was injected through the patella ligament into the right knee joint to induce arthritis (Davis & Perkins, 1993) (*n*= 6). Controls were uninjected (*n*= 6).

The knee joint diameter was measured with callipers across the knee joint before CFA injection and every 2 days thereafter for 7 days after CFA injection. Animals were killed at the peak of swelling and behavioural changes (3 days) (Wilson *et al.*, 2006; Kelly *et al.*, 2007) and dorsal root ganglia (DRG) removed for immunohistochemical staining for TRPA1.

The DRG were embedded in TissueTek (Sakura Finetek) and snap frozen. DRG (L4, ipsilateral to the arthritis) were sectioned in the transverse plane at 8 μm, thaw-mounted onto slides coated with aminopropyltriethoxysilane (Sigma-Aldrich) and processed for immunohistochemistry as previously described (Dumitrescu *et al.*, 2004), with some modifications. Sections separated by more than 50 μm were selected for processing, from the middle third of the DRG. Aldehydes are known to covalently bind to TRPA1, so sections were postfixed with Zamboni's fixative rather than 4% paraformaldehyde, as preliminary data suggested that paraformaldehyde fixation resulted in poor TRPA1 staining. Sections were incubated overnight with a primary antibody raised against the N-terminus of TRPA1 (generous gift of Prof. Jaime Garcia-Anoveros, Northwestern University Medical School, Chicago, IL, USA; previously characterized against rodent DRG; Nagata *et al.*, 2005) at 4°C at a dilution of 1 : 100. Slides were then extensively washed in phosphate-buffered saline, incubated with horseradish peroxidase-conjugated secondary antibody and the signal visualized using diaminobenzidine. Control sections were processed without the primary antibody. The percentages of TRPA1-expressing cells were calculated by counting the numbers of positive and negative cells with cytoplasmic staining, as defined by the blinded operator (JPD), in non-serial secretions (to avoid double counting) from each animal, with reference to control sections that were not exposed to the primary antibody. We did not attempt to quantify cell size distributions (Chopra *et al.*, 2000). The negative control indicated that nuclear staining in both DRG neurones and satellite cells was non-specific. TRPA1-expressing cells were counted in each of two sections from four to six animals per group and mean values for each animal were calculated and used in subsequent statistical analysis. Because a defined stereological approach was not employed, the percentages of neurones calculated may represent a biased estimate of the actual numbers of TRPA1-positive neurones.

### Drugs

All drugs were obtained from Sigma-Aldrich except for capsaicin, which was obtained from Tocris (Bristol, UK). Sodium pentobarbital was obtained in its sodium salt form and was made up in house (60 mg/mL) to allow i.p. and i.v. injection for anaesthetic induction and maintenance respectively. Drugs for intra-arterial injection were delivered in 10% ethanol, 10% Tween 80 and 80% saline (cinnamaldehyde, capsaicin and capsazepine) or saline (ruthenium red) at the concentrations shown in the text.

### Data and statistical analysis

Data are expressed as detailed in the figure legends. All analysis was performed using prism v4 for Macintosh and Windows (Graphpad Software, San Diego, CA, USA). Where appropriate, data were tested for normality (Gaussian distribution) prior to further statistical analysis. Cinnamaldehyde-evoked activity and mechanical thresholds were analysed using non-parametric tests. All other data were compared using parametric tests.

The mechanical thresholds of primary afferents were compared using Friedman's one-way anova followed by Dunn's *post hoc* test. Cinnamaldehyde-evoked activity was quantified as the number of action potentials in 60 s post injection less the number of action potentials in 60 s prior to injection. A few units had low-level spontaneous activity and this method of quantification could sometimes generate low negative values if these units were not cinnamaldehyde sensitive. If this occurred, negative values were corrected to 0.

Cinnamaldehyde-evoked activity in naive and CFA-injected rats was compared using a Mann–Whitney *U*-test. The effects of the transient receptor potential channel antagonists, ruthenium red and capsazepine, on cinnamaldehyde-evoked activity were compared using the Mann–Whitney test. The activity evoked by sequential cinnamaldehyde injections was compared using the Friedman one-way anova followed by Dunn's *post hoc* test.

Thermal thresholds were compared using a one-way anova followed by a Bonferroni *post hoc* test. Joint diameters at day 3 in naive and CFA-injected rats were compared using a Student's t-test. The proportion of cinnamaldehyde-responsive and cinnamaldehyde-unresponsive afferents in the two groups of rats was compared using a Fishers exact test. The numbers of TRPA1-immunoreactive cells were compared using a one-way anova followed by a Dunnett's *post hoc* test.

## Results

To determine whether TRPA1-positive afferent properties are affected in acute inflammation, specifically in relation to mechanical stimuli, the mechanical thresholds of TRPA1-positive and TRPA1-negative afferents from both inflamed and normal skin were obtained. TRPA1-positive afferents were identified using cinnamaldehyde (80 mm), a selective TRPA1 agonist (Bandell *et al.*, 2004), administered by close intra-arterial injection. This evoked robust activity in a population of primary afferent fibres (TRPA1-positive). Cinnamaldehyde applied over a longer period of time resulted in more prolonged activity (data not shown).

**Fig. 1:**
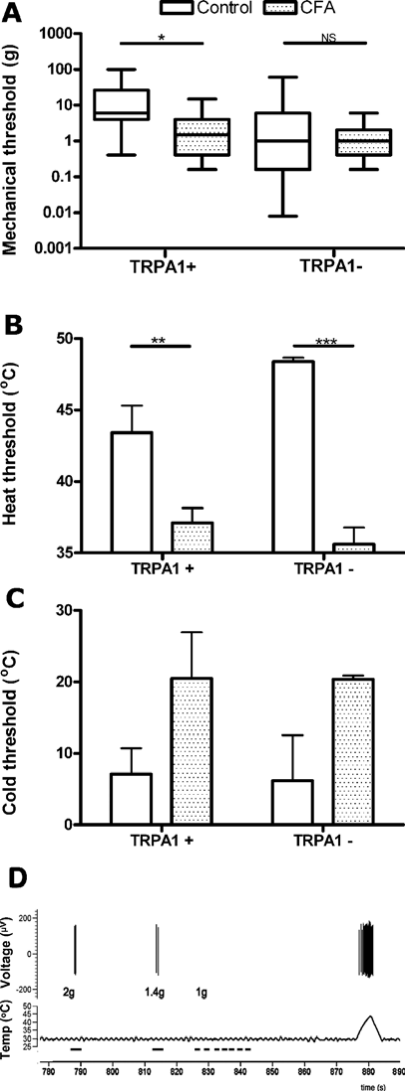
Activation thresholds in primary afferents in normal and inflamed animals. (A) Mechanical thresholds of the TRPA1-positive and TRPA1-negative afferents in normal (*n*= 55 units, 38 animals) and inflamed (*n*= 17 units, 8 animals) animals. Thresholds were significantly decreased in inflammation only in the TRPA1-positive population (19 normal, 10 inflamed). [Box and whisker plots show the 25th and 75th centiles (box), median (central line) and range (whiskers)] (*P* = 0.01). NB: Log scale on *y*-axis. (B) The heat thresholds for both TRPA1-positive (6 normal, 11 inflamed) and TRPA1-negative (6 normal, 4 inflamed) polymodal nociceptors are significantly lowered in inflammation (mean ± SEM; *P* = 0.005). (C) Cold thresholds were increased in C-fibre polymodal nociceptors in the TRPA1-positive and TRPA1-negative populations (2 to 3 normal, 2 to 3 inflamed; mean ± SD). (D) A representative digitized data trace of evoked activity in a single polymodal unit innervating inflamed skin. The dashes in the lowest trace denote the time of the mechanical stimuli, the intensity of which is given next to the elicited response (action potentials μV, top trace). Repeated stimulation with 1 g von Frey hairs (825–840 s) elicited no responses, unlike 1.4 and 2 g hairs (∼815 and 790 s, respectively). The middle trace shows the interface temperature of the Peltier device, which increases from 30 to 45 °C (beginning at ∼875 s), evoking a response at ∼35 °C. NS, not significant. *P < 0.05, **P < 0.01, ***P < 0.001.

The TRPA1-positive afferents innervating normal skin had significantly higher mechanical thresholds than TRPA1-negative units (Table 1, Fig. 1A, *P* < 0.01), indicative of TRPA1 expression on nociceptive afferents. All of the TRPA1-positive units were nociceptors. Some polymodal units had mechanical thresholds of < 1 g; these all had thermal thresholds in the noxious range, as previously reported (Lynn & Carpenter, 1982; Willis & Coggeshall, 2004). Only two units were Aδ afferents, with the rest being either polymodal nociceptors or mechanonociceptive C-fibre neurones. In inflamed skin the mechanical thresholds of the TRPA1-positive units were significantly lower than in the normal animals (Table 1, Fig. 1A, *P* < 0.01), whereas there was no difference in threshold in the TRPA1-negative units in inflammation (*P* = 0.2). Interestingly, when the primary afferents were not separated into TRPA1-positive or TRPA1-negative and the mechanical thresholds of all of these units were compared between normal and inflamed animals, there was no significant difference between the two groups (Table 1).

**Table 1:** Summary of the properties of the afferents studied, including the properties of the different subpopulations of TRPA1-responsive (TRPA1-positive) and TRPA1 non-responsive (TRPA1-negative) afferents

			Cold threshold (°C)			
	Mechanical threshold (g)	Heat threshold (°C)	C-fibres	Aδ-fibres	Afferents responding (%)	Evoked activity# (AP's in 60 s)
All units studied						
Control	2 (0.6–10)	45.9 ± 1.2	6.8 ± 1.8	18.2 ± 2.6	36	–
(*n*)	(55)	(12)	(5)	(3)	59	–
CFA	1 (0.4–3)	36.8 ± 0.8***	20.5 ± 4.6**	–	60	–
(*n*)	(17)	(15)	(5)	–	20	–
TRPA1-positive units						
Control	6 (4–26)††	43.4 ± 1.9††	7.13 ± 3.6	18.9 ± 3.4	–	10 ± 1
(*n*)	(19)	(6)	(3)	(2)	–	(42)
CFA	1.5 (0.4–4)*	37.1 ± 1.0**	20.5 ± 6.5	–	–	25 ± 5**
(*n*)	(10)	(11)	(3)	–	–	(30)
TRPA1-negative units						
Control	1 (0.16–8)	48.4 ± 0.3	6.2 ± 6.4	17.2	–	–
(*n*)	(36)	(6)	(2)	(1)	–	–
CFA	1 (0.4–2)	36.0 ± 1.2***	20.4 ± 0.5	–	–	–
(*n*)	(7)	(4)	(2)	–	–	–

Mechanical thresholds are given as median threshold (quartiles) and heat and cold thresholds as given as mean ± SEM (SD for groups where *n*≤ 3). Heat and cold thresholds are the temperature at which firing was evoked. Note that cold thresholds are only given for those mechanoreceptors in which cold responses could also be evoked (see text).

# Firing rates (mean ± SEM) are those evoked by intra-arterial cinnamaldehyde and thus these data can only be obtained from TRPA1-positive afferents.

* *P* < 0.05

** *P* < 0.01

*** *P* < 0.001,compared with the control in thesame group (pooled, TRPA1-positive or TRPA1-negative, i.e. the data 2 rows above);

†† *P* < 0.01, compared with the same condition in the other subgroup (e.g. control TRPA1-positive vs. control TRPA1-negative); one, two or three symbols denote the degree of significance, i.e. *P* < 0.05, *P* < 0.01 and *P* < 0.001. –, no units found/studied; AP, action potential.

To extend the understanding of the functional properties of TRPA1-positive afferents, we also examined the thermal thresholds in these mechanically-sensitive afferents. In contrast to the mechanical thresholds, which were significantly higher in normal animals than the TRPA1-negative afferents, the TRPA1-positive heat-responsive polymodal C-fibres had significantly lower heat thresholds than the TRPA1-negative C-fibres in normal animals (Table 1, Fig. 1B, *P* < 0.001). In the very few mechanocold afferents found (which are known to be present in only small numbers; Willis & Coggeshall, 2004), there were no obvious differences in cold thresholds between the TRPA1-positive and TRPA1-negative polymodal C-fibres from normal animals (Table 1 and Fig. 1C). The C-fibre polymodal units had cold thresholds clearly in the noxious range (7 ± 2°C) and these were significantly lower than those of the Aδ-fibre mechanocold-sensitive units (Table 1). Data from these units are therefore shown separately. No Aδ cold-sensitive units were identified in inflamed animals.

A comparison of the heat thresholds of cutaneous afferents in normal and inflamed skin revealed that the heat thresholds of all fibres studied were significantly reduced in inflammation (Table 1, *P* < 0.0001). Analysed separately, the heat thresholds of both TRPA1-positive and TRPA1-negative afferents in inflamed animals were significantly lower than those in normal animals (Table 1; Fig. 1B, *P* = 0.006, anova) but the thresholds in the pharmacologically defined populations were not significantly different from each other in inflammation. Similarly, the cold thresholds for the C-fibre units were significantly higher in inflammation than in normal animals. Although data are shown for the cold thresholds of TRPA1-positive and TRPA1-negative units, it was not possible to make direct comparisons between the groups due to the low numbers found (Table 1 and Fig. 1C). These data show that all mechanothermal units were sensitized to thermal stimuli in inflammation, irrespective of TRPA1 expression.

**Fig. 2:**
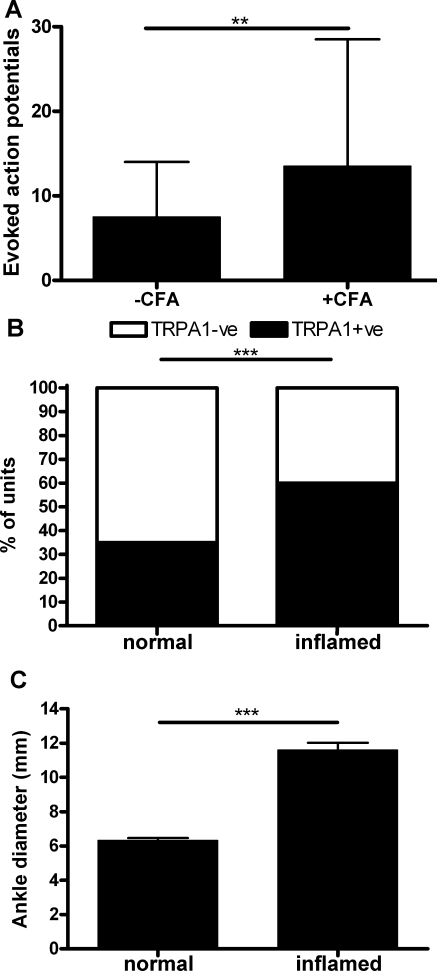
Primary afferent characteristics in CFA-induced inflammation. (A) CFA significantly enhanced cinnamaldehyde-evoked activity (number of action potentials) in afferents innervating inflamed skin (*n*= 42) more than in afferents innervating normal skin (*n*= 30; *P* < 0.01) (medians and interquartile range). (B) Functional TRPA1 responses could be evoked in 36% of fibres in normal animals (*n*= 59) and this was increased to ∼60% (*n*= 20) after CFA inflammation (*P* < 0.001). (C) Ankle joint diameters in normal and inflamed rats at 3 days after CFA injection. CFA injection caused significant swelling of the joints at this time (*n*= 4; *P* < 0.001). **P < 0.01, ***P < 0.001.

In addition to a reduction in the mechanical threshold of the TRPA1-positive afferents, inflammation almost doubled the amount of activity evoked by cinnamaldehyde (Fig. 2A). In naive animals, cinnamaldehyde evoked 8 (3 to 14) action potentials (median 25th–75th percentile) but after inflammation this increased to 14 (8 to 29) (Table 1; Fig. 2B, *P* < 0.01). Furthermore, the proportion of surveyed fibres that responded to cinnamaldehyde (80 mm) was also increased (Fig. 2B). In naive animals 36% of A-δ- and C-fibre afferents were TRPA1-positive, whereas this increased to 60% following inflammation (Fig. 2B, *P* < 0.001). This change in the proportion and sensitivity of TRPA1-positive afferents was associated with clear swelling in the area of the receptive fields under study (Fig. 2C).

**Fig. 3:**
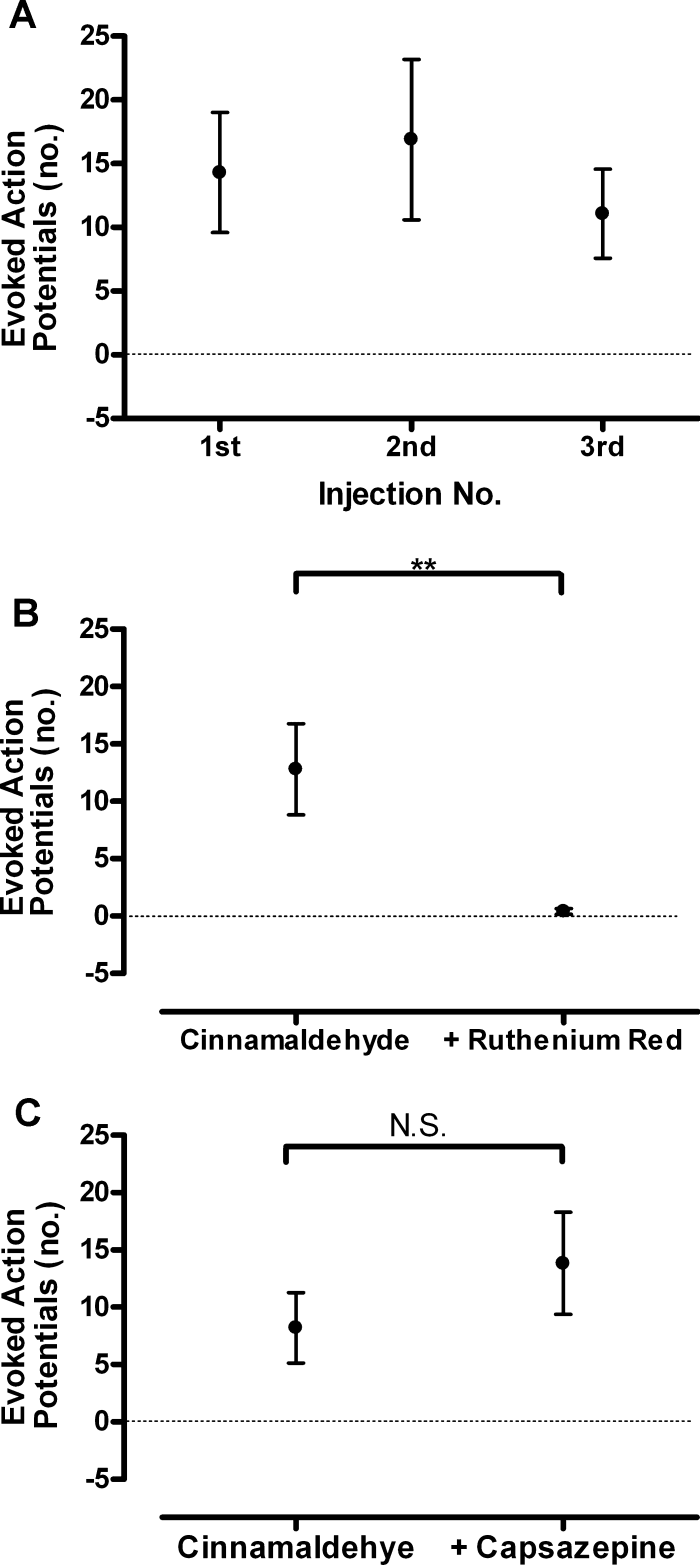
Pharmacological characterization of agonist and antagonist responses at TRPA1-positive primary afferents. (A) Response to three sequential doses of cinnamaldehyde (80 mm) does not desensitize with an interstimulus interval of 5 min (*n*= 27 units from naive and inflamed rats). (B) Ruthenium red significantly attenuated intra-arterial 80 mm cinnamaldehyde-evoked activity (mean number of action potentials and SEMs, *P* < 0.01, *n*= 5 units). (C) Capsazepine (500 μm) did not affect activity evoked by intra-arterial 80 mm cinnamaldehyde (means and SEMs, *n*= 6 units). NS, not significant. **P < 0.01.

Cinnamaldehyde (80 mm) elicited a robust burst of action potentials in responsive primary afferent fibres that did not show desensitization on repeated administration (Fig. 3A). This response was totally abolished by the calcium channel blocker ruthenium red (1 mm) (Fig. 3B, *P* < 0.01) but was unaffected by the TRPV1 antagonist capsazepine (500 μm) (Fig. 3C). The cinnamaldehyde-evoked activity in the presence of capsazepine was also not different from the cinnamaldehyde-evoked response in another group of afferents (compare Fig. 3A and C) but it should be noted that others have also reported a trend towards enhanced TRPA1 agonist responses after the application of capsazepine (Kosugi *et al.*, 2007). This dose of capsazepine has been previously shown to inhibit the capsaicin-evoked flexor reflex *in vivo* (Ando *et al.*, 2004). The complete lack of inhibition by capsazepine, in conjunction with the complete block by ruthenium red, can be interpreted as demonstrating that cinnamaldehyde is not evoking this response through activation of TRPV1 receptors. Ruthenium red is a non-competitive non-specific transient receptor potential channel blocker; in contrast, capsazepine is a TRPV1/transient receptor potential channel M8 antagonist (Bevan *et al.*, 1992; Weil *et al.*, 2005) with no known action at TRPA1 (Bautista *et al.*, 2005; Kosugi *et al.*, 2007). Blockade of these selective agonist-evoked responses by ruthenium red but not capsazepine has been used previously to indicate TRPA1-mediated events in the absence of commercially available TRPA1 antagonists (Bautista *et al.*, 2005; Kosugi *et al.*, 2007).

17 TRPA1-positive afferents were also studied for responsiveness to the TRPV1 agonist capsaicin. Additionally, 21 capsaicin-sensitive afferents were tested for cinnamaldehyde sensitivity; 94% of the TRPA1-positive afferents also responded to capsaicin (10 μm), whereas 76% of the capsaicin-sensitive afferents responded to cinnamaldehyde (80 mm). Of the nine C-fibre TRPA1-positive afferents also tested for heat responses in normal animals, six were activated by heat (mean threshold 43.4 ± 1.9°C). In inflammation, all TRPA1-positive C-fibres were heat responsive, with significantly lower thresholds (37 ± 1°C).

### Expression of TRPA1-like immunoreactivity in DRG neurones from acutely inflamed rats

**Fig. 4:**
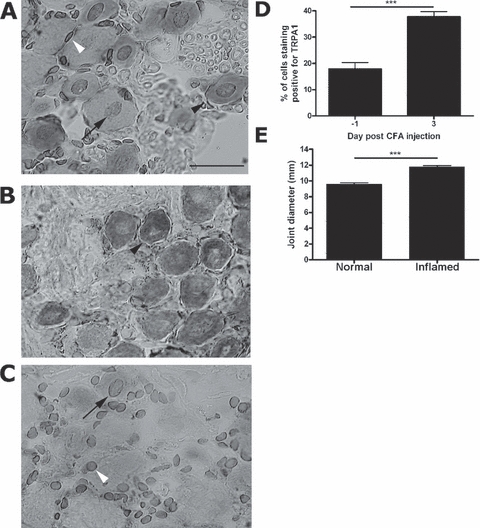
The number of DRG neurones showing TRPA1-like immunoreactivity is increased in acute inflammation. (A and B) TRPA1-like immunoreactivity was evident in small but not large neuronal cell bodies (black arrowhead) in sections from rat L4 DRG from control (A) and day 3 CFA (B) animals. Scale bar, 50 μm. (C) Omission of primary antibody resulted in loss of staining in neurones but not in satellite cells (white arrowhead) or DRG nuclei (black arrow), suggesting that this nuclear staining was non-specific. (D) The proportion of TRPA1-immunoreactive neurones increased in rat L4 DRG ganglia 3 days after CFA injection (*P* < 0.0.01, *n*= 4–6 per group). (E) Knee diameters were also significantly increased by CFA treatment 3 days after CFA injection (*P* < 0.001, *n*= 6 per group). ***P < 0.001.

In order to determine a possible mechanism through which an increased number of afferents might respond to the TRPA1 agonist cinnamaldehyde, we examined the expression of TRPA1 protein in L4 DRG neurones in animals with acute inflammation. In agreement with the increase in the number of afferents that responded to cinnamaldehyde, the percentage of DRG neurones expressing TRPA1 immunoreactivity was significantly increased 3 days after CFA injection (Fig. 4B and D, *P* < 0.001). This increase in TRPA1 expression was concomitant with CFA-induced swelling of the knee (Fig. 4E, *P* < 0.001).

## Discussion

In this study, we investigated how the properties of TRPA1-expressing afferents change in inflammation, with particular reference to mechanical and thermal thresholds and agonist-evoked responses. In inflammation, the mechanical activation thresholds of only TRPA1-expressing neurones decreased, whereas the thermal thresholds altered to a similar degree in both TRPA1-expressing and non-TRPA1-expressing afferents. TRPA1-positive afferents were sensitized to both agonist and physiological stimuli in inflammation. Our findings also show that TRPA1 is restricted to a subpopulation of nociceptive afferents, all of which are mechanosensitive, some of which are heat sensitive but very few of which are cold sensitive. These data support and extend previous histochemical (Story *et al.*, 2003; Jordt *et al.*, 2004; Kobayashi *et al.*, 2005; Nagata *et al.*, 2005) and functional studies on TRPA1-expressing afferents (Schmidt *et al.*, 1995; Diogenes *et al.*, 2007).

The TRPA1-positive afferent population comprises C-fibre high-threshold mechanoreceptors and polymodal receptors, and so was not restricted to polymodal nociceptors as we initially hypothesized. Polymodal nociceptors encode information on noxious mechanical, heat and cold stimuli, are usually responsive to inflammatory mediators, and their properties are often dramatically affected in inflammation (Andrew & Greenspan, 1999; Schaible *et al.*, 2006). Importantly, in inflammation, the mechanical thresholds of the TRPA1-positive afferents were significantly lowered compared with normal animals, whereas the thresholds of TRPA1-negative afferents did not change. During inflammation the thresholds of the TRPA1-positive cells fell to values similar to TRPA1-negative units in non-inflamed rats. These lowered thresholds, together with the recent findings of Petrus *et al.* (2007), imply that the inflammatory mechanical allodynia is mediated through a change in the properties of TRPA1-positive primary afferents. In contrast, however, Obata *et al.* (2005), in elegant studies using intrathecal TRPA1 antisense, did not find an effect of TRPA1-positive knockdown on inflammatory mechanical hyperalgesia. The reason for the disparity between these results is not clear.

Lowered mechanical thresholds could come about through increased sensitivity of TRPA1-positive mechanoreceptors and/or *de-novo* TRPA1 expression in afferents that have lower mechanical thresholds. The number of DRG neurones expressing TRPA1 increases in inflammation (these data; Obata *et al.*, 2005) so there is evidence to support the latter mechanism. There is no increase in cell size of TRPA1 mRNA-expressing cells after CFA (Obata *et al.*, 2005), indicating that this increased expression does not occur in large A-fibre, low-threshold neurones. With regard to the mechanical thresholds of individual afferents, it is thought that the mechanical thresholds of fine cutaneous primary afferent nociceptors are not affected by inflammation (Andrew & Greenspan, 1999; Koltzenburg, 1999) and behavioural changes in the mechanical withdrawal thresholds are attributed to central effects. Indeed mustard oil, a TRPA1 agonist, applied to skin lowered the mechanical thresholds of high-threshold dorsal horn neurones (Woolf *et al.*, 1994). In contrast, a lowering of the afferent mechanical thresholds is accepted as contributing to behavioural allodynia in arthritis (Schaible *et al.*, 2006). There are reports of lowered mechanical thresholds in primary afferent neurones of different species following inflammation or tissue damage (Cooper *et al.*, 1991; Davis *et al.*, 1993; Randich *et al.*, 1997; Yamashita *et al.*, 1999; Pogatzki *et al.*, 2002), administration of inflammatory mediators (Martin *et al.*, 1987, 1988; Ahlgren *et al.*, 1997; Chen *et al.*, 1999) and in arthritis (Guilbaud *et al.*, 1985; Schaible & Schmidt, 1985; Grigg *et al.*, 1986; Grubb *et al.*, 1991). In contrast, others report no change in rat (Reeh *et al.*, 1986; Kirchhoff *et al.*, 1990; Koltzenburg *et al.*, 1999; Schlegel *et al.*, 2004) and human (Olausson, 1998). Potential differences between reports that find no change in mechanical threshold and our work are not immediately obvious but could include the site of inflammation, superficial/cutaneous or deeper tissues, and *in vitro* vs. *in vivo* preparations. We saw lowered mechanical thresholds only in the TRPA1-expressing afferents, in agreement with other reports where changes in mechanical thresholds were only seen in some, but not all, C-fibres (Randich *et al.*, 1997; Yamashita *et al.*, 1999). Where afferent responses are pooled, as they are in most studies, any lowered threshold in a specific subpopulation, such as TRPA1-positive, could be missed. There is, in addition, the possibility that altered thresholds may not be observed, due to the nature of the stimulus applied using hand-held von Frey hairs (e.g. application of incremental rather than continuous mechanical stimuli and accurate repositioning of filaments for repeated stimulation). These potential methodological weaknesses did not, however, influence our ability to detect changes in the mechanical threshold in inflammation.

Inflammation also resulted in an enhanced response to cinnamaldehyde, showing agonist sensitization of TRPA1 receptors in inflammation, and an increased number of responsive neurones. These findings would suggest that the chemosensitivity of TRPA1-positive afferent fibres is enhanced in inflammation. Thus, where endogenous inflammatory aldehydes, such as 4-hydroxynonenal (Trevisani *et al.*, 2007) or other inflammatory mediators that sensitize TRPA1, are increased [such as protease activated receptor 2 (Dai *et al.*, 2007) or bradykinin (Bandell *et al.*, 2004; Bautista *et al.*, 2006)], more activity could be evoked than in uninflamed tissues. The combination of the sensitization and increased numbers of TRPA1 C-fibre afferents would lead to increased nociceptive input to the spinal cord in inflammation.

The majority of TRPA1-positive afferents also responded to capsaicin, in both normal and inflamed rats, indicating an almost 100% functional overlap of TRPA1 and TRPV1. Unsurprisingly, inflammation resulted in a lowered heat threshold in all of the afferents studied. The surprising finding with respect to heat was that in normal animals TRPA1-positive afferents had significantly lower heat thresholds than the TRPA1-negative population. Given that there are functional interactions between TRPA1 and TRPV1 *in vitro* and *in vivo* (Bandell *et al.*, 2004; Bautista *et al.*, 2006; Jeske *et al.*, 2006; Akopian *et al.*, 2007; Ruparel *et al.*, 2007), it is interesting to speculate that a TRPA1/TRPV1 interaction may be responsible for the lower heat thresholds seen in the polymodal C-fibres that express TRPA1 in comparison to those that do not. That both TRPA1-positive and TRPA1-negative afferents developed lowered heat thresholds in inflammation is probably attributable to the sensitization of TRPV1 in inflammation, which is present in afferents of both groups (these data; Kobayashi *et al.*, 2005) and is known to contribute to heat sensitization of afferents in inflammation (Davis *et al.*, 2000; Story *et al.*, 2003).

Although we identified a large number of tonically active C-cold afferents (Hensel & Zotterman, 1951), none of these were activated by cinnamaldehyde. In addition, in these fibres cold thresholds are difficult to determine as they encode temperature through dynamic changes in firing pattern rather than onset of firing at a specific temperature (Hensel & Zotterman, 1951), and these were therefore not included in these analyses. We identified very few TRPA1-positive cold-activated C-fibres, so interpretation of a possible role of these afferents in cold nociception is very limited. The threshold of the cold-responsive C-fibre afferents was raised in inflammation, indicating the development of a cold allodynia mediated through changes in primary afferent properties, consistent with reported behavioural changes (Obata *et al.*, 2005). Due to the low number of TRPA1-positive cold-responsive afferents identified we cannot draw definite conclusions on the possible role of TRPA1 in C-fibre nociceptors in inflammatory cold allodynia. Interestingly, we also identified a few TRPA1-positive cold-sensitive Aδ units in normal animals. Aδ but not C-fibres are particularly important in cold behavioural changes in nerve injury and icilin-evoked responses are enhanced in neuropathic rats (Kosugi *et al.*, 2007). It is possible that further study of TRPA1 in A-fibres, many of which are cold-responsive at very low stimulus temperatures (Simone & Kajander, 1997), may reveal a contribution of this class of fibres to the behavioural changes seen in inflammation (Obata *et al.*, 2005).

The aim of this study was to determine how the properties of TRPA1-expressing neurones are changed in inflammation *in vivo*. We identified an increase in the proportion of TRPA1-positive afferents, an increase in agonist sensitivity and lowered mechanical thresholds only in the TRPA1-positive fibres in inflammation. Together, these changes in primary afferents may contribute to enhanced afferent activity in inflammatory states.

## Abbreviations

CFA: complete Freund's adjuvant
DRG: dorsal root ganglia
TRPA1: transient receptor potential channel A1 (also known as ANKTM1)
TRPA1-negative: expressing no functional responses to TRPA1 agonist
TRPA1-positive: expressing functional responses to TRPA1 agonist
TRPV1: transient receptor potential channel V1 (also known as VR1)
